# Structural stability and energetics of grain boundary triple junctions in face centered cubic materials

**DOI:** 10.1038/srep08692

**Published:** 2015-03-03

**Authors:** I. Adlakha, K. N. Solanki

**Affiliations:** 1 School for Engineering of Matter, Transport, and Energy, Arizona State University, Tempe, AZ

## Abstract

We present a systematic study to elucidate the role of triple junctions (TJs) and their constituent grain boundaries on the structural stability of nanocrystalline materials. Using atomistic simulations along with the nudge elastic band calculations, we explored the atomic structural and thermodynamic properties of TJs in three different fcc materials. We found that the magnitude of excess energy at a TJ was directly related to the atomic density of the metal. Further, the vacancy binding and migration energetics in the vicinity of the TJ were examined as they play a crucial role in the structural stability of NC materials. The resolved line tension which takes into account the stress buildup at the TJ was found to be a good measure in predicting the vacancy binding tendency near the TJ. The activation energy for vacancy migration along the TJ was directly correlated with the measured excess energy. Finally, we show that the resistance for vacancy diffusion increased for TJs with larger excess stored energy and the defect mobility at some TJs is slower than their constituent GBs. Hence, our results have general implications on the diffusional process in NC materials and provide new insight into stabilizing NC materials with tailored TJs.

Nanocrystalline (NC) materials (mean grain size, *d* < 100 nm) often have enhanced mechanical properties compared to coarse-grained materials (*d* > 1 μm). Therefore, NC alloys are very attractive for multiple engineering applications including load-bearing structures^1^. The challenge with broader applicability of NC materials has been the stability of the non-equilibrium microstructure during processing and deformation. The structural stability, mechanical behavior, and fracture of NC materials is often driven by grain boundaries (GBs, planar defects), triple junctions (TJs, line defects), and their underlying structures^2^,^3^. Hence a fundamental understanding of the relationship between the linear/planar defect structures and the associated properties is important in the development of interface-dominant materials, especially in the case of NC materials where TJs constitute a large volume fraction^3^. Further, the role of TJ atomic structure on mass transport or diffusional processes, which directly controls the stability of NC materials, is a critical open question.

In NC materials, the grain growth behavior is typically characterized with the help of classical (2D) approaches such as the original von Neumann-Mullins relation (refer to further detail in Mullins)^4^. However, such a model provides an inadequate description of the grain growth behavior in NC materials as it ignores TJ contributions on the evolution of the grain growth behavior. In fact, Czubyako et al.^5^ experimentally showed that a low TJ mobility can induce a significant drag effect on the grain growth and subsequently proposed a modified von Neumann-Mullins relationship that, to some extent, captures the interplay between the GB and TJ kinetics on the grain growth behavior^5^. Nevertheless, such models are derived based on geometrically constrained TJs and do not account for the varying TJ structures to accurately capture the intrinsic role of TJs on the grain growth behavior of NC materials^6^,^7^,^8^,^9^. On the other hand, a number of groups have linked the structural stability of TJs to thermodynamic variables, such as the excess free energy, the resolved line tension, and the resolved line force of TJs^6^,^10^,^11^. For example, Gottstein et al. have shown that the excess Gibbs energy at the TJ can serve as a measure of the TJ mobility^6^. However, there is a lack of systematic investigations exploring various thermodynamic properties of TJs formed by commonly observed GBs^12^,^13^,^14^,^15^,^16^ and their links to the structural stability of NC materials, i.e., defect migrations.

In this work, for the first time, we present a systematic investigation using molecular statics along with the nudge elastic band (NEB) method^17^ to quantify the intrinsic properties for a range of special TJs (these TJs have been shown to occur frequently^12^,^13^,^16^) in Al, Cu, and Ni (see Table S1). The choice of materials studied in this manuscript highlights a large variation in the elastic and plastic anisotropies. The elastic anisotropy ratio, 
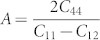
, for Al, Ni, and Cu is 1.16, 2.5, and 3.19, respectively, indicating that the Al is highly isotropic as compared to Ni and Cu. Similarly, the ratio between the stable and unstable stacking fault energies 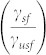 can be used to comprehended the plastic behavior of these materials, i.e., the ratio describes the competition between a full versus partial dislocation^18^. For instance, Al has the highest ratio between the stable and unstable stacking fault energies (0.83) followed by Ni (0.34) and Cu (0.28)^19^,^20^,^21^. Thus, these metals show a wide variation in critical elastic and deformation properties providing a broad platform to analyze the general behavior of TJs. For example, the role of TJ atomic structure on the vacancy binding and migration energetics was examined in the present manuscript as the vacancies can serve as an effective means for relieving the accumulated strain energy at the TJ^22^. However, when concentration of vacancies in the bulk exceeds the equilibrium concentration, it leads to an increase in the Gibbs free energy and produces a reactive thermodynamic force. This thermodynamic force provides resistance to the TJ motion during grain growth.

Finally, the relationship between the measured intrinsic properties and the structural stability was examined in detail. The vacancy binding energetics and the migration behavior is directly related to the structural stability of TJs^22^. Hence, it is critical to understand the intrinsic role between TJ thermodynamic properties and vacancy binding/migration characteristics. Therefore, our results have general implications on the diffusional/transport process in NC materials and provide new insight into stabilizing NC materials with tailored TJs through patterning^23^ or GB engineering^24^.

## Results

### Triple junction atomic structure

**Figure 1:**
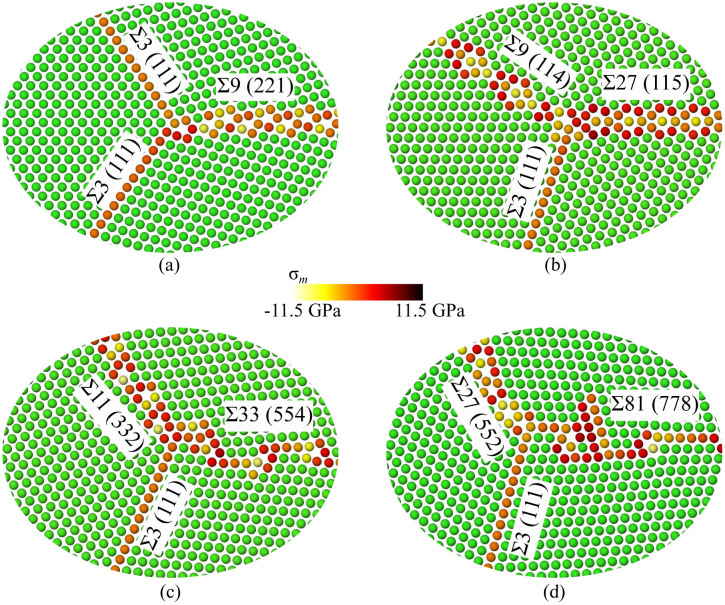
The minimized atomic configuration of: (a) Σ3-Σ3-Σ9 TJ, (b) Σ3-Σ9-Σ27, (c) Σ3-Σ11-Σ33, and (d) Σ3-Σ27-Σ81 TJs in Al. Atoms in a perfect fcc lattice are depicted as green while the defected atoms along the GB and the TJ are colored according to the variation in hydrostatic stress.

First, we focus on characterizing the TJ atomic structures by measuring the hydrostatic stress field and the net change in atomic volume due to the formation of TJs. These structural quantities provide a basis for measuring the TJ intrinsic properties, such as the excess energy, the resolved line tension and the resolved line force. For instance, the hydrostatic stress field is further used to compute the resolved line tension acting at the TJ, and the measured change in volume is directly proportional to the TJ misfit strain. Figure 1 shows the atomic hydrostatic stress field for a few (Σ3-Σ3-Σ9, Σ3-Σ9-Σ27, Σ3-Σ11-Σ33, and Σ3-Σ27-Σ81) TJs in Al highlighting the atomic arrangement due to GBs intersecting at the TJ. Details related to the construction of various TJs are presented in the methods section. The hydrostatic stress accumulated near the Σ3-Σ3-Σ9 TJ (Figure 1a) was higher when compared to the stress along the GBs. This clearly highlights that in the case of the Σ3-Σ3-Σ9, the TJ has a greater influence on the stress accumulation. On the other hand, in the case of the Σ3-Σ9-Σ27, the constituent GBs along with the terminating GB structures at the TJ have a large effect on the accumulated stress. In the case of the Σ3-Σ27-Σ81 (Figure 1d), an intrinsic stacking fault (ISF) was formed in the vicinity of the TJ. The localization of large stresses along the terminated GB structures can be attributed to the formation of the ISF, and further, the ISF can act as another defect sink.

**Figure 2:**
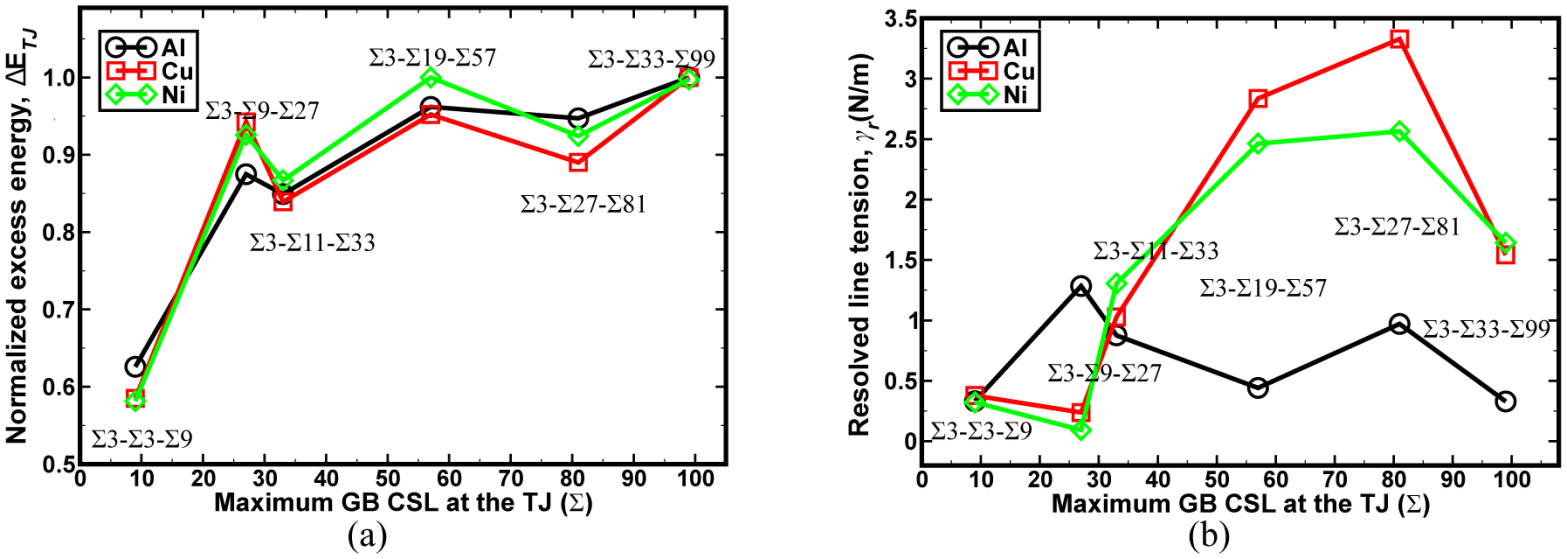
(a) The normalized excess energy of TJs in Al, Cu, and Ni. A clear trend can be observed indicating that the relative structural stability of the TJ was similar regardless of the chosen material type. (b) The resolved line tension acting along the TJ for Al, Cu, and Ni.

The net change in volume due to the formation of the TJ was also computed using the following relation to comprehend the misfit strain induced during the formation of a TJ.
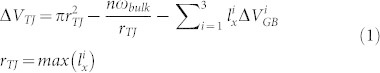
Here, *r_TJ_* is the radius for the TJ for calculating net change in volume, n is the number of atoms lying with a distance of *r_TJ_* from the TJ, *ω_bulk_* is the atomic volume in a pristine fcc lattice at 0 K, and 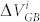 is the volume expansion along the GB plane per unit of the GB area for the *i*^th^ interface, and 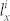 is the length of the unit vector tangential to the GB plane. The net change in normalized volume for all the metals follows no clear trend except that TJs with high Σ GBs undergo greater volumetric expansion in contrast to other TJs, see Figure 2a (also refer to Tables S2, S3, and S4). This suggests that the high Σ GBs would form a more diffused TJ. A similar trend was reported for TJs in Si using atomistic simulations^25^. In general, the volume change for Al TJs was found to be greater than those of the Cu and Ni. This variation is directly related to the differences in the lattice parameters of these metals (*a_Al_* > *a_Cu_* > *a_Ni_*).

### Characterizing triple junction thermodynamic properties

The total excess energy due to the formation of the TJ was computed with respect to the energy of the pristine fcc lattice. The excess energy due to the formation of TJs was found using the following relation:
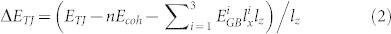
where *E_TJ_* is the energy of the defected structure with TJ and GB interfaces, *E_coh_* is the cohesive energy of each atom at 0 K in a pristine fcc lattice, and 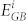 is the GB formation energy per unit area for the *i*^th^ interface. The net excess energy was normalized by the line length (*l_z_*) of the TJ. These results were found by the summation of atomic energies within a distance of 19 nm from the TJ. The trends for normalized excess energy for all the metals studied were found to be comparable (Figure 2a). For a few TJs studied in the open literature using atomistic methods^12^,^25^,^26^ and experimental work^27^, the excess energy computed here is in close agreement. The excess energy for TJs in Al formed by low energy GB interfaces was found to be higher when compared to the TJs with high energy interfaces. A similar trend was reported for TJs in Si using atomistic simulations^25^.

Next, we evaluate the resolved line tension which has been shown as a driving force for the TJ mobility during microstructural evolution^5^,^6^,^8^,^9^,^10^. The resolved line tension was computed using the relationship described in the method section. The variation in the resolved line tension trend (Figure 2b) correlates with the elastic anisotropy of metals studied, i.e., Cu (*A_Cu_* = 3.19) has the highest variation in the resolved line tension followed by Ni (*A_Ni_* = 2.5) and Al (*A_Al_* = 1.16). The relative stability of TJs based on the excess energy and the resolved line tension for the Σ3*^n^*-Σ3-Σ3*^n^*^−1^ TJs was found to be lower than that of Σ3*^n^*-Σ3-Σ3*^n^*^+1^ TJs (Figures 2a and 2b). Similar conclusions have been drawn in previous studies on TJs in Ni and other fcc materials^28^,^29^,^30^. The resolved line force acting at the TJ was computed with the help of the excess energies and the spatial arrangement of the intersecting GBs at the TJ. There was no clear trend observed between the resolved line force for the three materials and other intrinsic quantities (Table S2–4).

### Correlating the triple junction thermodynamic properties to vacancy binding energetics

**Figure 3:**
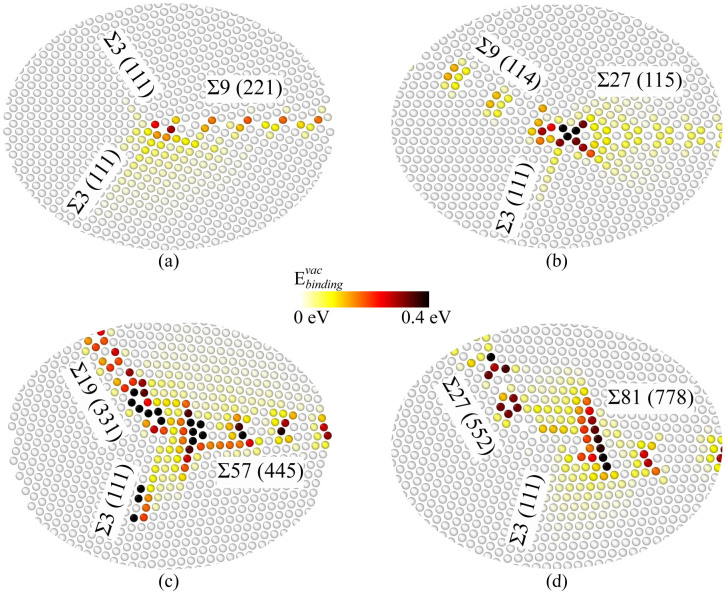
The atomic variation in vacancy binding energy near (a) Σ3-Σ3-Σ9, (b) Σ3-Σ9-Σ27, (c) Σ3-Σ19-Σ57, and (d) Σ3-Σ27-Σ81 TJs in Al. The atoms were colored based on the vacancy binding energy at each site. White atoms correspond to the bulk binding energy (~0 eV) and black atoms represent the maximum vacancy binding energy of 0.4 eV.

The vacancy binding energy in the vicinity of the TJ (≤4 nm) was investigated, see Figure 3. The vacancy binding energy (*E_b_*) is essentially the extra energy required to form a defect at a specific site near the defect region (in this case the TJ, 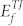) in comparison to the bulk formation energy of the defect (
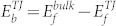
, where 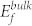 for Al, Cu, and Ni was 0.71, 1.29, and 1.64 eV respectively, refer to Refs. 31, 32 and Supplementary material). The effect of TJs on the binding tendency for atomic sites within the grain was found to be negligible, i.e., the atoms further away from the TJs are colored white (0 eV vacancy binding energy), indicating that there is no energy difference over the bulk lattice binding energy (see Figure 3). There is a clear difference in the numbers of preferred binding sites (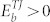) in the vicinity of the TJ between the Σ3-Σ3-Σ9 and Σ3-Σ9-Σ27 TJs (Figure 3a and 3b). The Σ3-Σ9-Σ27 TJ has preferred vacancy sites concentrated in a very small region around the TJ (3 Å radially) indicating the extent of the TJ elastic strain field. On the other hand, the Σ3-Σ19-Σ57 and Σ3-Σ27-Σ81 TJs have more stable vacancy sites (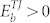) away from the TJ along the constituent GBs and along the ISF formed during the minimization of the TJ (Figures 3c and 3d).

**Figure 4:**
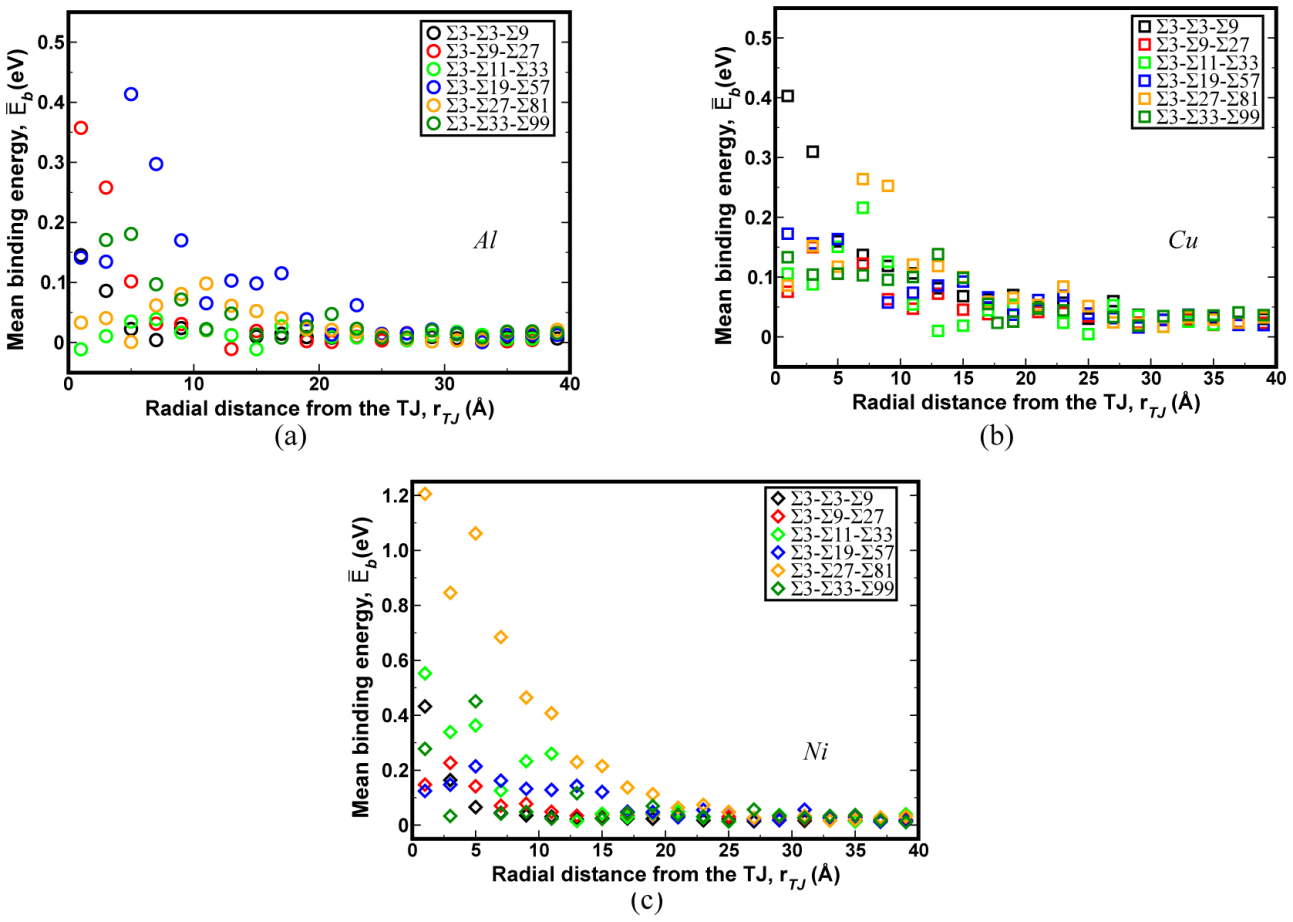
The mean vacancy binding energy as a function of radial distance from the TJ for (a) Al, (b) Cu, and (c) Ni. A total of 20 concentric bins were defined (*r_i_*_−1_ ≤ *r* ≤ *r_i_*) up to 4 nm from the TJ. In the cases of Al and Ni, the maximum binding energy was observed for TJs with the highest resolved line tension clearly suggesting that higher energy TJs were favorable sites for the vacancy binding.

Furthermore, the role of constituent GBs and the TJ on vacancy binding was investigated with the help of the mean binding energy as a function of radial distance from the TJ (Figure 4). The vacancy binding energy for a majority of TJs converged to the bulk vacancy formation energy at a length of approximately 2 nm from the TJ. In all three fcc metals studied, the vacancy binding energy was maximum (i.e. < 10 Å radially) for the TJs that had the maximum resolved line tension (Figure 2b). Nonetheless, there were a few exceptions such as the Σ3-Σ19-Σ57 Al and Σ3-Σ3-Σ9 Cu that also showed lower binding energy near the TJ. The reason for the observed lower binding energy for the Σ3-Σ19-Σ57 Al was due to the greater influence of GBs over the TJ.

### Vacancy migration along the triple junction line

**Figure 5:**
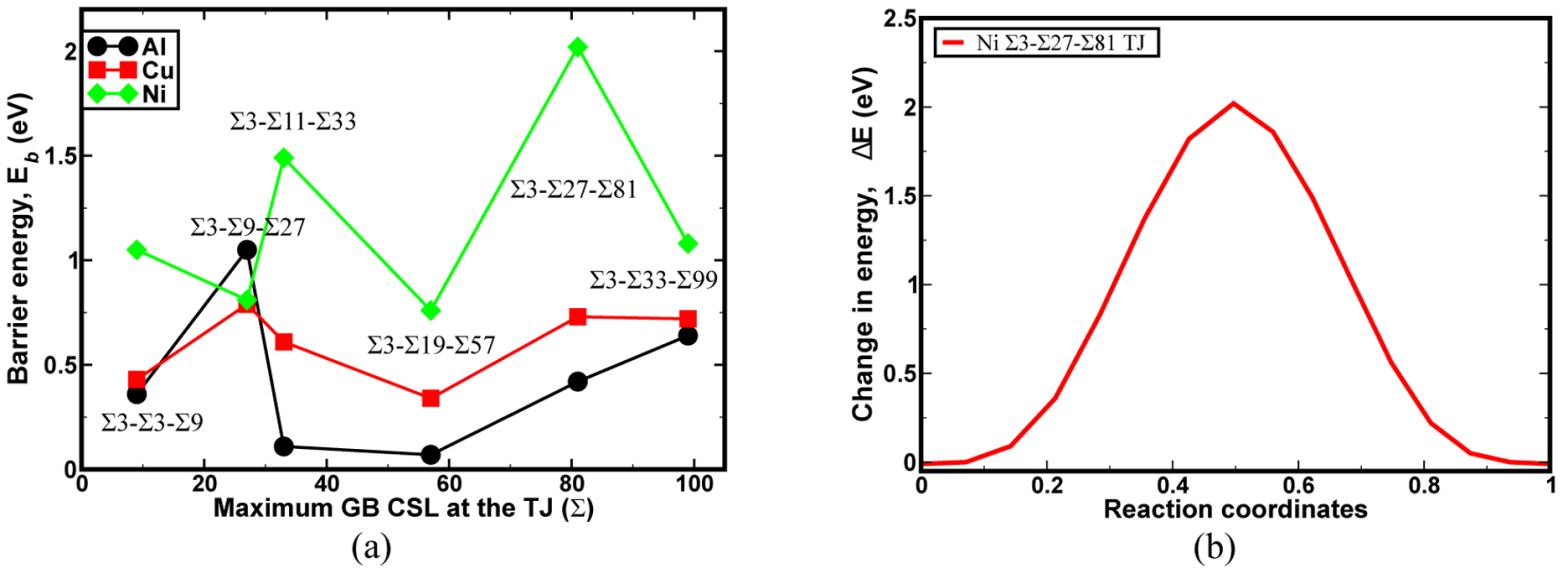
(a) The vacancy migration energies for Al, Cu, and Ni along the TJ. (b) The change in energy as the vacancy migrates in the Ni Σ3-Σ27-Σ81 TJ. In the case of Al and Cu, the migration energies trend correlates with the excess energies. In general, the vacancy migration energies of Ni TJs were much higher than that of Al and Cu TJs. The possible explanation for this trend can be attributed to the higher vacancy binding observed within the TJs plane in Ni.

Finally, using the NEB method, the role of TJ local structure on the kinetics of vacancies, i.e., migration energy within the TJ plane (Figure 5a) was characterized. The vacancy migration energy for both Al and Cu follows a similar trend. In general, the vacancy migration energy along the TJs in Ni was clearly greater than its counterparts in Al and Cu. The high vacancy migration energy in Ni TJs can be attributed to the availability of a large number of stable sites for vacancy binding near the TJ, as shown in Figure 4c. The Σ3-Σ27-Σ81 TJ in Ni was found to have migration energy of 2 eV (Figures 5a–b). The high migration energy can be explained with the help of the binding energy for the TJ (Figure 4c). The presence of extremely stable binding (~1.2 eV) sites for vacancies around the Σ3-Σ27-Σ81 TJ (Figure 4c) require far more energy to enforce vacancy migration (Figure 5b), clearly indicating a drag effect on the defect migration.

The effect of the interplay between a GB and the TJ on the grain growth has been discussed in previous work^5^,^9^. For example, it has been shown that below a certain critical grain size, the immobile TJs can create a drag effect to the microstructural growth. Here, we characterize the mass transport properties of the TJs by quantifying the activation energy for diffusion, i.e., the sum of vacancy formation and migration energies for various TJs (refer to Table S5).
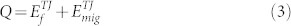
where 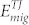 is the vacancy migration energy in the vicinity of the TJ.

**Figure 6:**
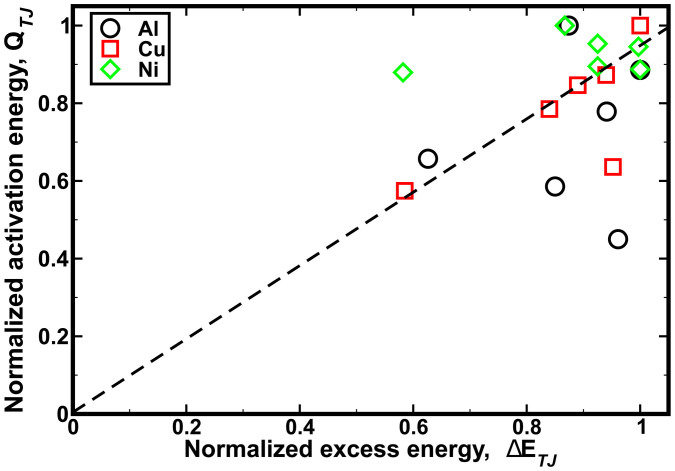
The normalized activation energy for diffusion in Al, Cu, and Ni TJs was plotted as a function of normalized excess energy. The extent of correlation between these thermodynamic quantities for various metals can be summarized as Cu ≫ Al > Ni (Ni was largely insensitive to the change in excess energy stored at the TJ).

The normalized activation energy for vacancy diffusion was plotted against the normalized excess energy due to formation of the TJs (Figure 6). In the case of Cu TJs, there was a strong correlation between the activation energy barrier and the excess energy. Conversely, activation energy was weakly correlated to the excess energy for TJs in Al. The activation energy for TJs in Ni was found to be insensitive to the excess energy of the TJ.

## Discussion

As mentioned earlier, the microstructural stability is critical for broader applicability of NC materials. Therefore, it is critical to evaluate the role of TJs and their constituent GBs on the structural stability. In this regard, the effect of the interplay between the TJs and their constituent GBs on the physical properties of NC materials has, so far, been myopically categorized into: a) the TJ behavior/properties can be entirely described by their constituent GBs; b) the formation of a TJ due to termination of the interface causes a change in the behavior of one or more constituent GBs; and c) the properties of TJs are independent of their constituent GBs^33^. Thus, to account for this interplay, for the first time, the microstructural stability of various TJs with frequently observed GBs in fcc metals (Al, Ni, and Cu) have now been examined.

The quantification of TJ mobility in NC materials while incorporating the atomistic scale details is a challenging prospect with current experimental and modelling techniques. Further, the measurement of intrinsic structural and thermodynamic quantities of the TJ at the atomistic scale is critical as these quantities are thermodynamically related to the mobility of the TJ^6^,^10^,^11^. The hydrostatic stress field and the net volume change can be used to measure the degree of variability in the TJ local structural arrangement, which influences the mechanical behavior of NC materials. For example, the local TJ misfit strain is a function of the net volume change during the formation of the TJ, which can significantly alter the vacancy binding energetics (Figure 3). Further, the net volume change is directly related to the lattice parameter of the metals (Table S2–4), i.e., the largest volumetric changes were observed for Al followed by Cu and Ni TJs, respectively. Similarly, the variation in the atomic scale hydrostatic stress field of the TJ (Figure 1) indicates that the stress buildup at the TJ is not correlated with the GB coincident site lattice value (CSL) or GB energy. In other words, a TJ formed by intersecting GBs with high CSL values or GB energies can have a small hydrostatic stress distribution. These measured local structural properties, as highlighted here, are related to various thermodynamic quantities of the TJ, which significantly influence the stability of NC materials. For instance, the high hydrostatic stress distribution around the TJ can be relieved through the transport of vacancies. The magnitude of resolved line tension, which is a function of the hydrostatic stress field of the TJ, is related to that of the maximum vacancy binding tendency at the TJ (Figure 4). That is the TJs with higher resolved line tensions exhibit a large number of stable sites for the vacancy binding in the immediate vicinity of the TJ (Figure 3). Therefore, TJs with a higher resolved line tension can induce a significant drag on the mobility of the TJ with respect to its constituent GBs^5^. The interplay between the GB and TJ dominates the vacancy binding behavior. For instance, in a Σ3-Σ3-Σ9 Al TJ, the stable sites for binding were clustered near the TJ suggesting a strong influence of the TJ, Figure 3a. On the other hand, in the case of the Σ3-Σ19-Σ57 TJ, the stable sites were spread along the GB, implying that the GB structure and the relative position of the TJ to the GB structure are critical Figure 3c.

The flux of vacancies from the TJ to the bulk offers a faster method for relieving stored elastic energy results in the subsequent rearrangement of the GB network^22^. Hence, the excess energy measurement of the TJ is also intuitively appealing since it takes into account the TJ atomic structural arrangement and it is related to the TJ mobility. Moreover, as evident from Figure 2a, the magnitude of excess energy for the TJs 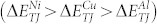 are inversely related to the lattice parameter of the material investigated here (*a_Al_* > *a_Cu_* > *a_Ni_*). In other words, a higher atomic density of the metal results in a lower dissipation of the excess energy during formation of the TJ. The excess stored energy during the formation of the TJ can be a realistic measure for predicting the structural stability of the GB network, see Figure 6. For instance, for Cu TJs, there is a strong correlation between the activation energy barrier and the excess energy, i.e., an increase in the excess energy results in a significant drag effect on the TJ mobility. In the case of Ni, the trends between the excess energy and activation energy are not correlated because of the high excess energies for all Ni TJs. Nonetheless, these findings are in agreement with previous works on the drag effect created by TJs during grain growth^7^,^9^,^22^,^34^.

In summary, for the first time, various intrinsic quantities of the TJ at the atomic scale were systematically quantified. Hence, this study provides generalized insights regarding the structural stability of NC materials. Moreover, these new atomistic perspectives provide a physical basis for recognizing the role between the TJ and vacancy transport in fcc materials. Since, both kinetic and thermodynamic properties of defects are very different at different triple junctions in different materials. Further, the defect mobility at some triple junctions can be slower than their constituent GBs. This is significant for applications where extreme environment damage generates lattice defects and TJs act as sinks for both vacancies and interstitial atoms. Further, our results have general implications on the diffusional/transport process in NC materials and provide new insight into stabilizing NC materials with tailored TJs through patterning^23^ and/or GB engineering^24^.

## Methods

**Figure 7:**
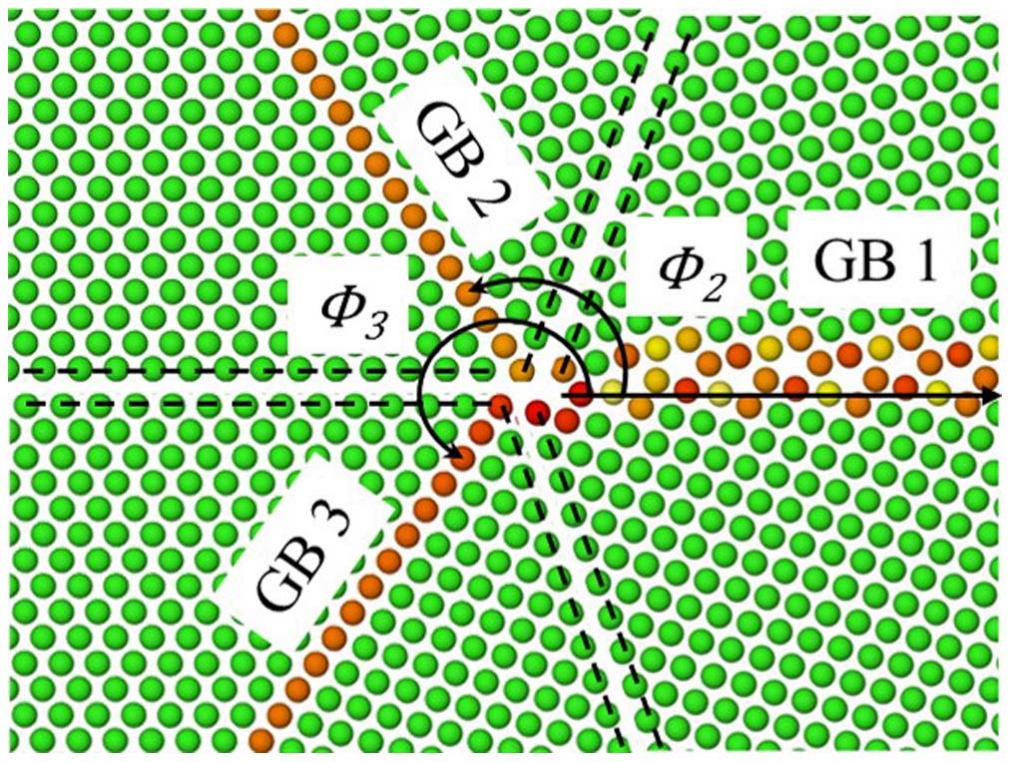
Schematic showing construction of a GB TJ configuration. The GBs were minimized separately and a wedge was carved out along the {001}/{011} plane represented by dashed lines.

Molecular statics simulations using LAMMPS^35^ were employed in this work to investigate the structural stability of TJs. The atomic interactions in this work were described using the embedded atom method (EAM) potential for Cu, Ni, and Al^19^,^20^,^21^. These EAM potentials were parameterized using an extensive database of energies and configurations from density functional theory calculations and have been used to accurately define different material behaviors such as surface energies, generalized stacking fault energies, etc. (e.g., Refs. 19,20,21). In this work, TJs were constructed using <110> symmetric tilt grain boundaries (GBs), see Table S1. These GBs were characterized using the structural unit method^36^,^37^. As with past work^31^,^32^,^38^,^39^, an atom deletion criterion, multiple initial configurations, and various in-plane rigid body translations were utilized to accurately obtain an optimal minimum energy GB structure via the conjugate gradient energy minimization process. The energy and force minimization convergence criterion was 1e-25. Circular wedges with a radius of 20 nm from TJ were then cut from the GBs along the stitch plane, i.e. {001}/{011} planes, see Figure 7^15^,^40^.

Subsequently, GBs labelled 2 and 3 were rotated by *ϕ*_2_ and *ϕ*_3_ about the GB tilt axis as shown in Figure 7. Lastly, the three wedges were brought together, the overlapping atoms were removed, and the energy minimization was carried out at 0 K. According to the Herring's relation the TJ attempts to minimize the resolved surface tension during formation. The resolved line tension acting at the TJ was approximated at the atomic scale using the following relation:
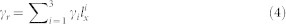
The surface tension for the intersecting GBs (*γ_i_*) was defined by averaging normal and tangential stresses acting over a region of ±20 Å normal to the interfaces and up to 19 nm from the TJ. The resolved line force was another quantity used to understand the energetics of TJs. This was defined as:
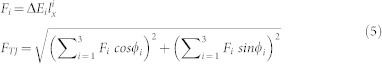
The excess energy per unit GB area (ΔE_i_) subsequent to the formation of the TJ was defined over a region of ±20 Å normal to the GB. The line force for all the GBs was resolved at the TJ (F*_TJ_*) to obtain the line force at the TJ.

The vacancy formation energy at an atomic site 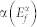 is defined as
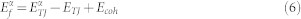
here *E_coh_* is the cohesive energy/atom in a perfect fcc lattice and 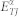 and *E_TJ_* are the total energies of the TJ simulation cell with and without the vacancy, respectively. It is useful to relate the vacancy formation energy in the TJ configuration 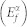 with that in the bulk 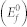 to define vacancy binding energy at an atomic site α, lying within 4 nm of the TJ
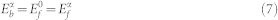
in order to provide a measure of energy required to move the vacancy from the TJ into the bulk or vice versa. The nudge elastic band method^17^ was employed to study the migration behavior of vacancies along the TJ line. The energy and force minimization criterions used for NEB calculations were 1e-25 and 1e-6, and 8 partitions/replications were used to find the minimum energy path for vacancy migration. The influence of temperature on the activation energy for vacancy migration can be taken into consideration with the help of transition state theory based methods as shown in previous studies^41^,^42^. Nevertheless, the qualitative findings observed for the intrinsic properties of the TJ and the activation energy would still be consistent at higher temperatures.

## Author Contributions

I.A. and K.N.S. equally contributed to this work. I.A. developed the necessary simulation techniques and performed all the calculations. All the authors discussed the results and wrote the manuscript.

## Supplementary Material

Supplementary Information supplementry text
