# Supplementary material for: Structural stability and energetics of grain boundary triple junctions in face centered cubic materials
Source: Sci Rep. 2015 Mar 3;5:8692. doi: 10.1038/srep08692 (PMC4346801; doi:10.1038/srep08692)
Supplement: Supplementary Information — supplementry text [file srep08692-s1.doc]

**Structural stability and energetics of grain boundary triple junctions in face centered cubic materials**

I. Adlakha, and K.N. Solanki^*^

*School for Engineering of Matter, Transport, and Energy, Arizona State University, Tempe, AZ*

^*^*(480)965-1869; (480)727-9321 (fax), E-mail: kiran.solanki@asu.edu, (Corresponding author)*

Table S1. Details of grain boundary triple junctions along with the CSL and the misorientation (*θ)* for each GB interface.

| **Triple Junction** | **GB #1 (*Σ, θ*)** | **GB #2 (*Σ, θ*)** | **GB #3 (*Σ, θ*)** |
| --- | --- | --- | --- |
| Σ3-Σ3-Σ9 | Σ9 (221), 141.06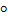 | Σ3 (111), 109.47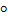 | Σ3 (111), 109.47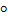 |
| Σ3-Σ9-Σ27 | Σ27 (115), 31.59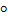 | Σ9 (114), 38.94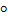 | Σ3 (111), 109.47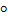 |
| Σ3-Σ11-Σ33 | Σ33 (554), 121.01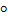 | Σ11 (332), 129.52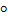 | Σ3 (111), 109.47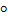 |
| Σ3-Σ19-Σ57 | Σ57(445), 97.05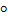 | Σ19 (331), 153.47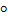 | Σ3 (111), 109.47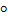 |
| Σ3-Σ27-Σ81 | Σ81(778), 102.12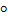 | Σ27 (552), 148.41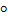 | Σ3 (111), 109.47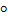 |
| Σ3-Σ33-Σ99 | Σ99(557), 90.58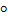 | Σ33 (441), 159.95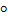 | Σ3 (111), 109.47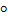 |

Table S2. Various intrinsic quantities such as the resolved line tension, the resolved line force, and the excess energies related to various TJs in Al.

| **Triple Junction** | **Volume Change,** 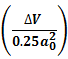 | **Line Tension,** 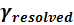 **(N/m)** | **Line Force,** 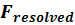 **(N)** | **Excess Energy due to TJ,**  **ΔE (J/m)** |
| --- | --- | --- | --- | --- |
| **Σ3-Σ3-Σ9** | 3.85 | 0.33 | 4.1 x 10^-6^ | 9.2 x 10^-9^ |
| **Σ3-Σ9-Σ27** | 8.24 | 1.28 | 4.1 x 10^-6^ | 1.3 x 10^-8^ |
| **Σ3-Σ11-Σ33** | 9.40 | 0.91 | 4.6 x 10^-6^ | 1.25 x 10^-8^ |
| **Σ3-Σ19-Σ57** | 3.65 | 0.44 | 1.0 x 10^-5^ | 1.45 x 10^-8^ |
| **Σ3-Σ27-Σ81** | 11.59 | 0.98 | 8.5 x 10^-6^ | 1.39 x 10^-8^ |
| **Σ3-Σ33-Σ99** | 9.69 | 0.33 | 9.7 x 10^-6^ | 1.47 x 10^-8^ |

Table S3. Various intrinsic quantities such as the resolved line tension, the resolved line force, and the excess energies related to various TJs in Cu.

| **Triple Junction** | **Volume Change,** 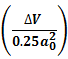 | **Line Tension,** 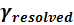 **(N/m)** | **Line Force,** 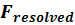 **(N)** | **Excess Energy due to TJ,**  **ΔE (J/m)** |
| --- | --- | --- | --- | --- |
| **Σ3-Σ3-Σ9** | 10.50 | 0.37 | 4.5 x 10^-6^ | 1.44 x 10^-8^ |
| **Σ3-Σ9-Σ27** | 6.95 | 0.24 | 6.5 x 10^-5^ | 2.32 x 10^-8^ |
| **Σ3-Σ11-Σ33** | 2.51 | 1.03 | 1.7 x 10^-4^ | 2.07 x 10^-8^ |
| **Σ3-Σ19-Σ57** | 4.19 | 2.83 | 5.2 x 10^-5^ | 2.35 x 10^-8^ |
| **Σ3-Σ27-Σ81** | 6.98 | 3.33 | 9.6 x 10^-5^ | 2.2 x 10^-8^ |
| **Σ3-Σ33-Σ99** | 10.13 | 1.54 | 1.3 x 10^-5^ | 2.47 x 10^-8^ |

Table S4. Various intrinsic quantities such as the resolved line tension, the resolved line force, and the excess energies related to various TJs in Ni.

| **Triple Junction** | **Volume Change,** 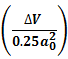 | **Line Tension,** 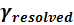 **(N/m)** | **Line Force,** 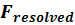 **(N)** | **Excess Energy due to TJ,**  **ΔE (J/m)** |
| --- | --- | --- | --- | --- |
| **Σ3-Σ3-Σ9** | 9.89 | 0.27 | 1.2 x 10^-6^ | 2.68 x 10^-8^ |
| **Σ3-Σ9-Σ27** | 6.45 | 0.09 | 1.8 x 10^-5^ | 4.02 x 10^-8^ |
| **Σ3-Σ11-Σ33** | 6.22 | 1.34 | 9.2 x 10^-6^ | 3.77 x 10^-8^ |
| **Σ3-Σ19-Σ57** | 2.90 | 2.66 | 1.5 x 10^-5^ | 4.35 x 10^-8^ |
| **Σ3-Σ27-Σ81** | 5.44 | 2.56 | 2.6 x 10^-5^ | 4.02 x 10^-8^ |
| **Σ3-Σ33-Σ99** | 8.28 | 1.52 | 1.5 x 10^-5^ | 4.34 x 10^-8^ |

Table S5. The activation energy for vacancy migration along the TJ, Q*_TJ_* (eV) for various TJs investigated in Al, Cu and Ni.

| **Triple Junction** | **Activation energy along the TJ, Q*_TJ_* (eV)** | | |
| --- | --- | --- | --- |
|  | **Al** | **Cu** | **Ni** |
| **Σ3-Σ3-Σ9** | 0.92 | 1.31 | 2.26 |
| **Σ3-Σ9-Σ27** | 1.4 | 1.99 | 2.3 |
| **Σ3-Σ11-Σ33** | 0.82 | 1.79 | 2.57 |
| **Σ3-Σ19-Σ57** | 0.63 | 1.45 | 2.28 |
| **Σ3-Σ27-Σ81** | 1.05 | 1.93 | 2.45 |
| **Σ3-Σ33-Σ99** | 1.24 | 2.28 | 2.43 |
